# Patient-reported experience with hypoglossal nerve stimulation in the treatment of obstructive sleep apnea

**DOI:** 10.1007/s11325-023-02887-0

**Published:** 2023-08-05

**Authors:** M. Braun, M. Wollny, C. Schoebel, J. U. Sommer, C. Heiser

**Affiliations:** 1 Department of Pneumology, University Medicine Essen – Ruhrlandklinik, West German Lung Center, University Duisburg-Essen, Duisburg, Germany; 2 Faculty of Sleep and Telemedicine, University Medicine Essen – Ruhrlandklinik, West German Lung Center, University Duisburg-Essen, Duisburg, Germany; 3 MedImbursement, Tarmstedt, Germany; 4 Department of Otorhinolaryngology/Head and Neck Surgery, Klinikum rechts der Isar, Technical University of Munich, Munich, Germany; 5 ENT-Center Mangfall-Inn, Bad Aibling, Germany

**Keywords:** Patient-reported experience, Patient-centered research, Therapy perception, Hypoglossal nerve stimulation, Upper airway stimulation

## Abstract

**Background:**

Breathing-synchronized hypoglossal nerve stimulation (HNS) is routinely used as an alternative treatment for patients with obstructive sleep apnea (OSA). Significant and clinically relevant improvements in disease severity and OSA symptoms such as daytime sleepiness as well as overall quality of life have been reported in randomized-controlled trials and large real-world cohort studies. However, so far, few data exist on patient-reported experience with the treatment.

**Methods:**

A structured survey with 22 questions was constructed using five-level Likert scales (1 = no agreement, 5 = complete agreement) to evaluate patient experience with HNS and perception of the treatment in the domains “Overall experience with therapy,” “Experience with treatment process,” and “Side-effects from treatment.” Additional data were collected on current symptom status, measured with Epworth sleepiness scale (ESS) questionnaire, and OSA disease history. Multiple linear regression analysis was conducted to test associations of medical variables and response behavior. Correlations between variables and domains, as well as individual items, were assessed using Spearman rank test.

**Results:**

A total of 75 patients from Germany who were treated with breathing-synchronized HNS were enrolled (mean age 57.3 years, 78% male), and 71 questionnaires with complete data were included for analysis. Two-thirds of participants (67%) had a history of OSA history for 5 years or longer. Of all patients, 76% had normalized OSA symptoms at time of the study (ESS: 6.4 ± 5.0) and 98% reported using stimulation therapy every night. Regression analysis revealed an association of current symptoms measured with ESS and response behavior. Hence, patients with normalized daytime sleepiness reported significantly more positive experience across all domains assessed, compared to patients with residual daytime sleepiness. Overall, only 2% of participants reported side effects that made them reduce or discontinue stimulation therapy. The rate of reported side effects was associated with current symptom control under therapy.

**Conclusions:**

Overall patient-reported experience with breathing-synchronized HNS therapy was positive and high satisfaction with the treatment process was observed. Side effects occurred, but rarely affected subjective use of the therapy or satisfaction. Subjective experience and perception are influenced by residual daytime sleepiness with stimulation therapy.

## Introduction

Among sleep disorders, obstructive sleep apnea (OSA) is one of the most common diseases and affects up to 1 billion adults globally, of which up to 425 million having moderate-to-severe OSA [1]. OSA is characterized by nocturnal cessation of breathing due to collapse of upper airway soft tissues, which can lead to fragmentation of sleep from frequent arousals [2]. This sleep fragmentation can cause significant symptoms, ranging from daytime sleepiness, fatigue, and reduced productivity to mood disorders such as depression [3, 4]. The repeated disturbances in gas exchange in the lungs can cause oxygen desaturation during sleep which can lead to systemic inflammation [5]. If left untreated, OSA can lead to the development or worsening of cardiovascular, cerebrovascular, metabolic, and neurological comorbidities, such as coronary heart disease, hypertension, diabetes, and others [6]. Long-term studies have shown up to five times higher mortality in patients with OSA who are untreated or undertreated [7].

Major risk factors for OSA include obesity, male gender, advanced age, and alcohol abuse, smoking, or use of sedative medications [8]. Diagnostic evaluation for OSA consists of collecting subjective information using a validated questionnaire to assess severity of health-related quality of life, and recording sleep to determine the frequency and duration of obstructive and desaturating events [9]. To assess the severity of daytime sleepiness, which is often the leading symptom, the Epworth Sleepiness Scale (ESS) is the most commonly used [10]. The ESS is an eight-item questionnaire that has been validated in a number of different languages. In addition to its use in diagnostic assessment, the ESS is also widely used to evaluate the efficacy of interventions to treat OSA and is a common subjective endpoint in clinical trials [11].

Although a variety of therapies is now available to treat OSA, nightly positive airway pressure (PAP) is the most commonly used primary treatment because of its short implementation time, high efficacy, and low cost [12]. Beside PAP therapy, oral appliances that hold the mandible in an anterior position to open the retropalatal space, and various resecting surgeries are available for alternative treatment when PAP therapy is not tolerated. Hypoglossal nerve stimulation (HNS) was introduced more than a decade ago and is established as a safe and effective therapy for OSA in many healthcare systems [13, 14]. As is the case for other chronic diseases, long-term therapy acceptance is critical to achieve sustainable control of OSA and its symptoms as well as to avoid development or progression of complications and comorbidities. Evidence has emerged over the past years, that patients suffering from OSA have diverse preferences, and the attributes of treatments can be of different importance to patients [15]. This leads to varying demand for specific therapies and underlines the need for a diverse portfolio of OSA therapies, including novel options such as HNS [16, 17].

The evaluation of new health technologies increasingly includes the assessment of patient-reported outcomes. This often includes not only subjective outcomes in the form of patient-reported outcomes measures (PROMs) of an intervention or treatment that measure health-related quality of life, either generic or disease-specific, and comparison to baseline or other interventions [18]. However, more recently, patient-reported experience measures (PREMs) are used to measure perceptions of the care received and can be used to measure and benchmark the performance of healthcare processes [19]. PROMs are mainly used to identify subjective effects from a condition or from an intervention from the perspective of the affected individuals. In the context of health technology evaluation, PROMs are mainly used to compare changes of symptoms against either baseline or other health services, and their objective is to evaluate direct utilities derived from a particular intervention like a treatment or a diagnostic test, in the form of endpoints such as efficacy and effectiveness. PREMs on the other hand aim to generate additional information on utilities and disutilities of health services. This can be for example the perception of received care or the performance of healthcare services with regard to process quality of a treatment pathway, satisfaction with received treatments or interactions with the healthcare providers. PREMs can therefore generate important additional insights on health interventions beyond the direct outcome assessment. PREMs can help to understand the intervention better and uncover improvement opportunities on how care is delivered.

HNS therapy is based on breathing-synchronized activation of upper airway dilator muscles during sleep by mild electrical stimulation that is delivered from a fully implantable neurostimulator [20–22]. The treatment is highly effective in reducing OSA-events and improving symptoms, and is well accepted by patients. Though HNS therapy was evaluated in multiple clinical trials, limited evidence is available on the experience of treated patients, besides commonly reported subjective outcome measures. The aim of this study was thus to evaluate patient-reported experience with HNS treatment in subjects with OSA who could not adhere to PAP therapy.

## Methods

Based on research into the importance of various attributes of OSA treatments, a structured survey using Likert scales was developed to assess patient-reported experience with nocturnal stimulation therapy [15]. Twenty-two items were constructed to assess patient experience in the areas of overall experience with therapy, experience with the treatment process, and side effects, and participants were asked to indicate their agreement with declarative statements on five-point Likert scales (1 = strongly disagree, 5 = strongly agree). Of the twenty-two items used in the survey, thirteen had a positive direction, which means that high agreement with the statements indicates a positive experience (Table 1). Nine items, which were used to assess experience of side-effects, had a negative direction, which means that lower scores indicate a more positive perception.

**Table 1:** Domains of patient-experience included in study

Domain	Items	Direction
Overall experience with therapy	• I can sleep well with HNS therapy. • HNS therapy improves my daytime sleepiness. • HNS therapy improves my comorbidities. • I use HNS therapy every night. • I use HNS therapy all-night. • My sleep quality has improved with HNS therapy. • My snoring improved with HNS therapy. • HNS therapy reduces the problems I had due to OSA.	Negative to positive: 1 (fully disagree = *negative* experience) ➔ 5 (fully agree = *positive* experience)
Experience with treatment process	• I am satisfied with HNS therapy. • I am satisfied with the physicians at the HNS center. • I can use HNS therapy as expected. • HNS therapy improves my health. • HNS therapy improves my Quality of Life.	Negative to positive: 1 (fully disagree = *negative* experience) ➔ 5 (fully agree = *positive* experience)
Side-effects from therapy	• Implantation surgery was a burden for me. • I experienced pain after the implantation surgery. • I wake up from HNS therapy • I have side-effects from HNS therapy. • Due to side-effects, I use HNS therapy less often. • Side-effects make me stop HNS therapy during night. • The implantable pulse generator bothers me. • I am handicapped due to HNS therapy.	Positive to negative: 1 (fully disagree = *positive* experience) ➔ 5 (fully agree = *negative* experience)

Additional data were collected on demographics, OSA disease history and HNS treatment history. Current subjective outcomes of HNS therapy were assessed using the Epworth Sleepiness Scale [10].

Patients with OSA and intolerance to PAP therapy who received breathing-synchronized HNS therapy (Inspire™ Upper Airway Stimulation, Inspire Medical Systems, Inc., Golden Valley, US) and were using this treatment since at least six months were eligible and received the questionnaire after giving informed consent.

### Statistical analysis

The survey data were managed using the Statistical Package for Social Sciences (IBM, New York, USA). Results are presented as mean with standard deviation unless otherwise stated. Responses, and thus the perceived patient experience, were considered positive when the Likert-score was four or five in the domains with positive direction (Overall experience with HNS therapy and Experience with treatment process). Patient experience was considered negative when items were scored three or less in these domains. For the domain Side-effects from treatment, responses and thus patient experience were considered negative when there was high agreement with the respective items (Likert-score 4 or 5). A positive patient-experience was indicated by scores of three or lower in this domain.

Individual item and overall reliability were estimated by calculating Cronbach’s alpha. To compare group differences, Student’s *t*-test was used for nominal data and Mann-Whitney *U*-test for ordinally scaled data. Multiple linear regression analysis was performed separately for each domain to identify potential effects of medical variables on response behavior. Correlations between variables and domains, as well as between individual items, were assessed using the Spearman rank test. *P*-values <.05 were considered statistically significant for all tests used.

## Results

A total of 75 subjects were enrolled and received the questionnaire. Four participants had to be excluded due to incomplete answering of the questionnaires, which made 71 datasets (95%) available for analysis. Reliability of the items, calculated with Cronbach’s α, ranged from .732 to .947, which was considered acceptable.

Most were men (85%), had a history of OSA for five years or longer, and were using breathing-synchronized HNS for one year or longer (Table 1). The average ESS score, as measure of residual daytime sleepiness, was 7.1 ± 4.9 and 73% of participants had normalized ESS values (ESS <10).

### Overall experience with therapy

**Fig. 1:**
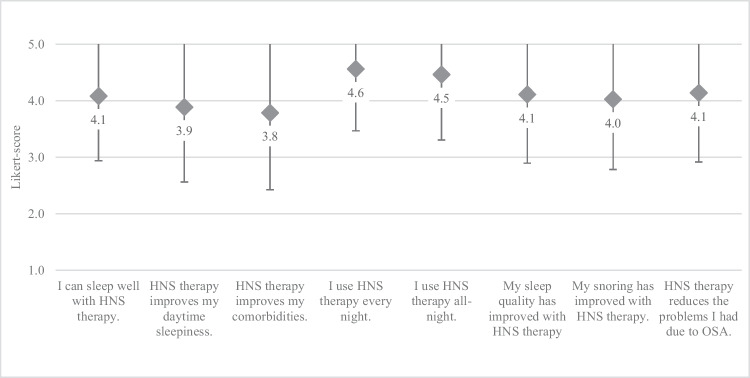
Likert-scores for domain “Overall experience with therapy” (mean Likert score ±SD; 1 – fully disagree, 5 – fully agree)

Experience with breathing-synchronized HNS therapy was reported positive with on average 77% positive answers (defined as a Likert score ≥ 4) in this domain, and a mean Likert-score of 4.1 ± 1.2 (Fig. 1). Subjective adherence was stated high with greatest agreement in this domain in the items “I use HNS therapy every night” and “I use HNS therapy all night” (4.6 ± 1.1 and 4.5 ± 1.2). High scores in the item “HNS therapy improves my daytime sleepiness” correlated with subjective improvement of snoring (r_s_= .809, *p* <.001). A high correlation was further found between the items “My sleep quality has improved with HNS therapy” and “HNS therapy reduces the problems I had due to OSA” (r_s_= .875, *p* <.001).

### Experience with treatment process

**Fig. 2:**
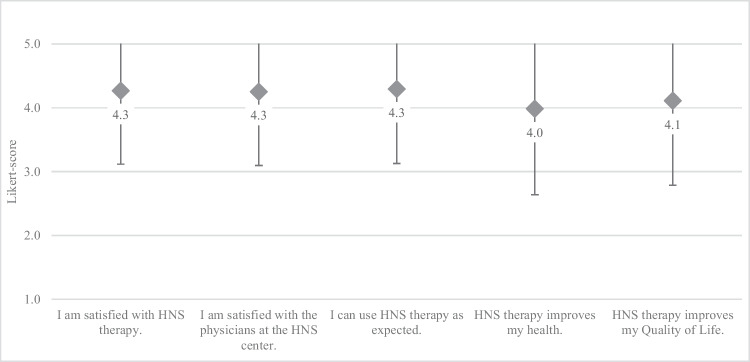
Likert-score for domain “Experience with treatment process” for initiation of breathing-synchronized HNS therapy (mean Likert score ± SD; 1 – fully disagree, 5 – fully agree)

Patient-reported experience with the care process in general was positive with a mean Likert-score of 4.2 ± 1.2 and on average 76% positive answers. The items “I am satisfied with HNS therapy, I am satisfied with the physicians at the HNS center” and “I can use HNS therapy as expected” showing Likert-scores of 4.3 ± 1.2 each, which were among the highest for the entire questionnaire (Fig. 2). Therapy satisfaction (I am satisfied with HNS therapy) and subjective improvement in quality of life (HNS therapy improves my Quality of Life) correlated highly (r_s_= .924, *p* <.001).

### Side-effects

**Fig. 3:**
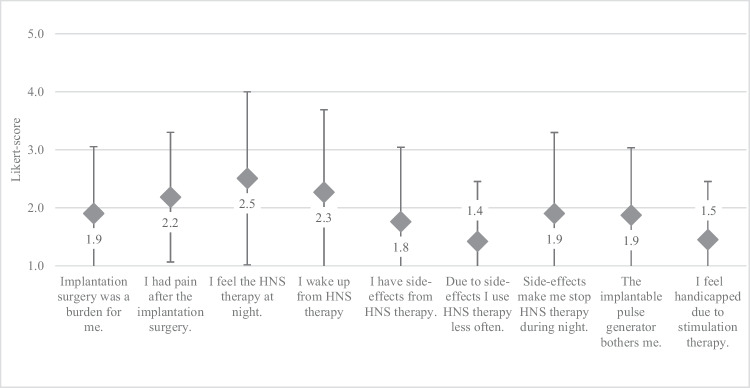
Likert-scores for domain “Side-effects from therapy” (mean Likert score ± SD; 1 –fully disagree, 5 – fully agree)

Perceived side-effects from therapy, which are relevant factors for long-term therapy adherence and patient satisfaction, and their impact on patient-reported experience were evaluated in this domain (Fig. 3). Overall, a mean agreement of 1.9 ± 1.0 was stated by participants in this domain, indicating little agreement with items evaluating the occurrence and severity of side-effects as well as potential consequences from them. Perceived perioperative morbidity was low with 8.5% of participants agreeing with the item “Implantation surgery was a burden for me” and 16% confirming “I had pain after the implantation surgery.”

Two items in this domain covered the most common side-effects with HNS therapy, which are sensation of stimulation during nightly use and awakening due to stimulation. While almost one third of participants in this study stated feeling HNS therapy at night (30%, mean Likert-score 2.5 ± 1.5), less report waking up from stimulation at night (21%, mean agreement 2.3 ± 1.4). These side-effects do not lead to lower subjective adherence overall, with only 6% reporting to use HNS therapy less often (mean Likert-score 1.4 ± 1.0) or discontinuing therapy at night (18.3%, mean agreement 1.9 ± 1.4). Overall impairment due to HNS therapy was low, with only 6% agreement in the item “I feel handicapped due to stimulation therapy” (mean Likert-score 1.5 ± 1.0). Greater agreement with this item correlated with the item “I feel HNS therapy at night” (r_s_= .796, *p* <.001), and the item “I wake up from HNS therapy” (r_s_= .782, *p* <.001). Furthermore, the items “Due to side-effects, I use HNS therapy less often” and “I feel handicapped due to HNS therapy” showed a high correlation (r_s_= .796, *p* <.001).

### Influence of medical variables on response behavior

To assess the influence of other variables on the three domains of patient-reported experience, five potentially relevant variables were tested in multiple linear regression analyses: ESS score at time of study participation, age, time since diagnosis, duration since HNS therapy initiation, and gender. ESS score and age had statistically significant influence on response behavior in all three domains, while gender was only significant in the domain “Experience with treatment process,” where female gender had a positive effect on agreement with the statements (Table 2). Since the effect of “age” was low in all domains (Coeff = -0.021, -0.021 and 0.022), “ESS score at time of study participation” as a measure of treatment success was selected for further testing (Coeff = -0.142, -0.084 and 0.098).

**Table 2:** Patient cohort

*N*	71
Age (mean, SD)	57.3 ± 10.1
Gender (%, male / female)	85 / 16
Recent ESS (mean, SD)	7.1 ± 4.9
Normalized ESS (%)	73
OSA history (%)	
1-2 years	7
2-5 years	38
5-10 years	24
> 10 years	31
HNS history (%)	
< 1 year	41
1-2 years	48
2-5 years	7
> 5 years	4

**Fig. 4:**
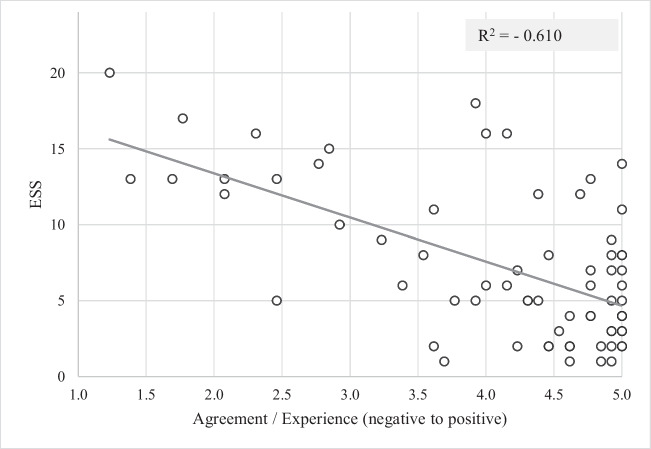
Correlation of current symptom status (Epworth Sleepiness Scale) and patient experience (mean Likert-score of items in the domains with positive direction (Overall experience with therapy and Experience with treatment process))

**Fig. 5:**
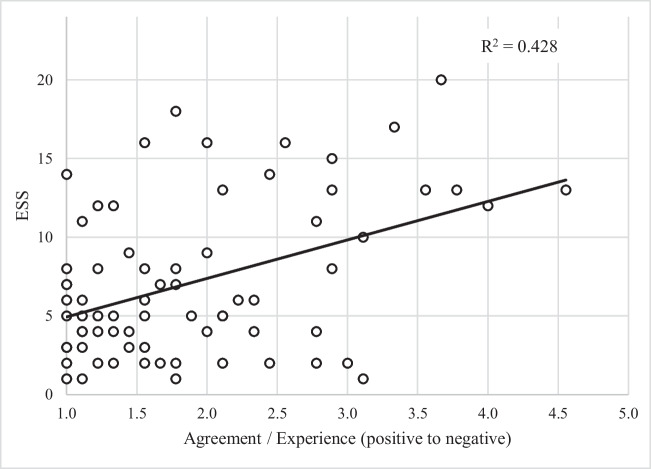
Correlation of current OSA symptoms (Epworth Sleepiness Scale) and patient experience (mean Likert-score of items in the domain with negative direction (Side effects from therapy))

In the regression analysis, a moderate negative correlation was found between the “ESS score at time of study participation” and the agreement with the sum score of items with a positive direction (R^2^ = -0.610), so lower ESS scores were associated with a more positive patient-reported experience (Fig. 4). For items with a negative direction, a low positive correlation between “ESS score at time of study participation” and sum score of the items was found (R^2^ = 0.428), which means that higher ESS scores correlated with greater agreement with statements on side-effects and thus a less positive patient experience (Fig. 5).

To assess the influence of subjective treatment efficacy on responses, participants were clustered into two subgroups contingent to their reported ESS scores. Patients with ESS values < 10 were considered having normalized daytime sleepiness under HNS therapy (*n*= 52, 73%), and those with values ≥ 10 were considered symptomatic from OSA (*n*=19, 27%). Across all domains, response profiles vary substantially depending on degree of subjective therapy efficacy (Table 3). Patients with normalized ESS values stated significantly higher agreement in all items in the domains “Overall experience with therapy and Experience with treatment process.”

**Table 3:** Results of linear regression analyses

	Coeff	SE	*t* Stat	*p-*value
Overall experience with therapy				
ESS score	-0.142	0.021	-6.881	< .001
Age	-0.021	0.010	-2.063	.043
Time since OSA diagnosis	-0.042	0.105	-0.400	.690
Time since therapy initiation	0.096	0.131	0.737	.464
Gender	-0.012	0.288	-0.042	.967
Experience with treatment process				
ESS score	-0.084	0.016	-5.327	< .001
Age	-0.021	0.008	-2.783	.007
Time since OSA diagnosis	-0.047	0.081	-0.577	.566
Time since therapy initiation	0.060	0.100	0.598	.552
Gender	0.471	0.213	2.216	.046
Side-effects from therapy				
ESS score	0.098	0.023	4.323	< .001
Age	0.022	0.011	2.034	.046
Time since OSA diagnosis	0.095	0.116	0.825	.412
Time since therapy initiation	0.058	0.143	0.407	.685
Gender	0.570	0.308	1.851	.069

In the domain “Side-effects from therapy,” scores were significantly higher for patients reporting higher ESS values, meaning greater presence of side-effects, with on average one point greater agreement on the Likert-scale. The only the exception were the items “The implantable pulse generator bothers me” (*p*= .108) and “Implantation surgery was a burden for me” (*p*= .421). Patients with higher ESS values also mentioned more often to stop therapy due to side-effects from stimulation, which aligns with the adherence items “I use HNS therapy every night*”* and “I use HNS therapy all night,” on which higher-ESS patients reported significantly lower agreement than those without residual daytime sleepiness (*p* = .010 and *p* = .008) (Table 4).

**Table 4:** Differences in response profiles between participants with and without normalized daytime sleepiness

Item	ESS < 10 (mean Likert score ± SD)	ESS ≥ 10 (mean Likert score ± SD)	*p*-value
Overall experience with therapy			
I can sleep well with HNS therapy.	3.1 ± 1.5	4.5 ± 0.7	.001
HNS therapy improves my daytime sleepiness.	2.5 ± 1.2	4.4 ± 0.9	<.001
HNS therapy improves my comorbidities.	2.8 ± 1.4	4.2 ± 1.2	.001
I use HNS therapy every night.	3.7 ± 1.7	4.9 ± 0.4	.010
I use HNS therapy all-night.	3.6 ± 1.7	4.8 ± 0.6	.008
My sleep quality has improved with HNS therapy.	3.2 ± 1.5	4.5 ± 0.9	.002
My snoring improved with HNS therapy.	2.9 ± 1.4	4.4 ± 0.9	<.001
HNS therapy reduces the problems I had due to OSA.	3.1 ± 1.5	4.5 ± 0.8	.001
Experience with treatment process			
I am satisfied with HNS therapy.	3.4 ± 1.5	4.6 ± 0.8	.005
I am satisfied with the physicians at the HNS center.	3.5 ± 1.3	4.5 ± 0.9	.004
I can use HNS therapy as expected.	3.7 ± 1.4	4.5 ± 1.0	.044
HNS therapy improves my health.	2.8 ± 1.6	4.4 ± 0.9	.001
HNS therapy improves my Quality of Life.	2.9 ± 1.7	4.5 ± 0.8	.001
Side-effects from therapy			
Implantation surgery was a burden for me.	2.1 ± 1.3	1.8 ± 1.1	.421
I experienced pain after the implantation surgery.	2.6 ± 1.0	2.0 ± 1.1	.035
I feel HNS therapy at night.	3.5 ± 1.5	2.2 ± 1.3	.003
I wake up from HNS therapy	3.3 ± 1.7	1.9 ± 1.1	.004
I have side-effects from HNS therapy.	2.6 ± 1.6	1.4 ± 1.0	.007
Due to side-effects, I use HNS therapy less often.	2.2 ± 1.6	1.2 ± 0.5	.017
Side-effects make me stop HNS therapy during night.	2.7 ± 1.7	1.6 ± 1.1	.022
The implantable pulse generator bothers me.	2.3 ± 1.4	1.7 ± 1.0	.108
I am handicapped due to HNS therapy.	1.7 ± 0.7	1.1 ± 0.4	.002

## Discussion

The aim of this study was to evaluate the experience of patients treated with breathing-synchronized HNS therapy, including their overall perception of the care they received from the hospitals during the treatment pathway and their experience of regular routine use after acclimatization. As this is a relatively new treatment option for patients with OSA, information on these aspects is important to fully understand the effects of this treatment and may help to inform patients considering HNS therapy, caregivers, and clinicians. This is the first study to comprehensively evaluate PREMs with breathing-synchronized HNS in a larger cohort. The study adds to previous research showing a generally high level of satisfaction with treatment, associated with significant reductions in OSA severity and improvements in patient-reported outcomes [20, 22–24]. PREMs were developed with the aim of being a useful adjunct to daily clinical practice at the individual patient level, to help focus attention on quality of life, and subsequently as an outcome measure for quality assessment.

The results of this study show a positive patient-reported experience with breathing-synchronized HNS therapy across all domains, with participants reporting significant improvements in their subjective health. This positive perception was independent of gender, age, time since OSA diagnosis, and time since initiation of HNS therapy. However, a positive association was found for the presence of daytime sleepiness at the time of the study as measured by the ESS questionnaire. This instrument was used in this study as a simple measure of treatment success and was associated with high levels of agreement with statements in the positive-poled domains of overall experience with therapy and experience with the treatment process. This demonstrates a relationship between subjective treatment effectiveness and patient-reported experience. Interestingly, a negative relationship of a similar magnitude was found for the domain with negative direction “Side-effects from therapy,” indicating that patients with residual symptoms of OSA are more likely to report side effects. In addition, the items “I can sleep well with HNS therapy” and “My sleep quality has improved with HNS therapy” showed significantly lower agreement in patients with residual symptoms, suggesting a link between patient experience of HNS therapy and the outcome of improvements in daytime sleepiness due to improved sleep quality. A similar relationship between patient-reported experience of side effects, such as awakening from stimulation, and patient-reported outcomes was reported by Hofauer et al. in an early analysis of patient experience with HNS therapy [25]. The potential negative impact of stimulus awakening on subjective outcomes was further confirmed in a study by Steffen et al., who found an association between insomnia and poorer patient-reported outcomes and significantly lower patient satisfaction [26]. Patients with insomnia were also more likely to report symptoms of depression, which in turn may affect patient experience. In addition to potential effects on sleep architecture, which may affect subjective experience of sleep quality, this may also be explained by a lower efficacy of stimulation leading to a lower reduction in snoring in patients with residual OSA. In this context, Pordzik et al. reported an inverse correlation between pre-operative insomnia levels and post-operative objective measures of OSA, highlighting the importance of insomnia for treatment success in patients undergoing HNS treatment [27].

For a new treatment such as breathing-synchronized HNS, the extent to which patient experience has been analyzed is remarkable, as the user perspective has long been absent from research into the treatment of sleep-disordered breathing. A recent meta-analysis of patient experience with PAP therapy identified data from only 25 studies with a total of 398 subjects, which is low given the time this treatment is in use and the scale of worldwide adoption [28]. The same is true for assessing patient experience with oral appliances, for which there is even less evidence. As objective measures of OSA and patient-reported outcomes assess only certain aspects of the overall success of a treatment, it would be valuable to assess patient-reported experience more often in regulated clinical trials as well as in real-world studies. Given the chronic nature of OSA, the patient’s experience of care pathways, diagnostic, and therapeutic processes is an important factor that may influence long-term adherence and thus the effectiveness of prescribed interventions.

### Limitations

This study has certain limitations that deserve attention. First, this study assessed only patients’ experiences at one point after the start of therapy, and the length of time that participants used HNS therapy varied. The data should therefore be interpreted with caution, as the experience may change over time in a more. It should also be noted that the experience in the process-related areas was linked to the structure of the German health care system and may not be transferable to other health care contexts with a different organization of health care provision. Certainly, the patient population enrolled in the study and their individual previous experiences also affected the results, and it is known from other interventions and indications that previous interactions with health care providers may affect responses [28, 29].

It is also important to note that although the results show a correlation between patient-reported outcomes and patient-reported experiences, they do not imply a causal relationship. However, it is likely that there is a close relationship between the experience of side effects, such as awakening from stimulation. As most patients receiving HNS today have symptomatic OSA, it is possible that incomplete reduction of daytime sleepiness may lead to lower satisfaction and therefore poorer patient experience.

## Conclusion

In this study, participants treated with breathing-synchronized HNS therapy, the patient-reported experience was positive, as indicated by high levels of agreement with therapy-related statements in the domains “Overall experience with therapy” and “Experience with the treatment process.” Patients reported low levels of agreement with statements in the domain “Side-effects from therapy,” meaning that patients were little affected by treatment related side-effects. Response-behavior was associated with current symptoms of daytime sleepiness as measured by the ESS questionnaire. Patients with normalized ESS scores reported significantly more positive experiences with HNS therapy.

## Data Availability

The data that support the findings of this study are not openly available due to reasons of sensitivity and are available from the corresponding author upon reasonable request.
